# Role of blue dye for sentinel lymph node detection in early endometrial cancer

**DOI:** 10.1186/s10397-017-1026-0

**Published:** 2017-11-29

**Authors:** Stefano Restaino, Carlo Ronsini, Angelo Finelli, Emanuele Perrone, Giovanni Scambia, Francesco Fanfani

**Affiliations:** 1 Gynecological Oncology, Hospital G. Bernabeo, Ortona, CH Italy; 2 Department of Medicine and Aging Sciences University “G. d’Annunzio” of Chieti-Pescara, Chieti, CH Italy; 3 Division of gynecological oncology, Department of Obstetrics and Gynecology, Catholic University of Sacred Heart, Rome, RM Italy

**Keywords:** Lymph node, Blue dye, Endometrial cancer

## Abstract

**Background:**

Sentinel Lymphonode analysis has become a barely new and innovative way to treat early stages of endometrial cancer (Ballester et al., Lancet Oncol 469–476, 2011; Buda et al., Ann Surg Oncol 2975-81, 2016). Indocyanine green cervical injection is considered gold standard for mapping nodes’ drainage. Blue dye is used as a valid alternative in many centers, due to the lower cost of execution. The objective of this video is to prove that methylene blue dye’s cervical injection is a valid and “low-cost” method to obtain mapping of lymphatic drainage in patient with early endometrial cancer.

**Methods:**

Fifty-four-year old women, with a recent diagnosis of endometrial cancer IA G2, we performed a radical Hysterectomy type A. We diluted in equal proportions the blue dye and saline and injected 1 cl in depth and 1 cl on the surface of the cervix, at 3 o’clock and 9 o’clock. After 20 min, it was shown with precision the lymphatic drainage until the first lymph node station from both sides.

**Results:**

One external iliac lymph node and one obturator were resected on the left side and one external iliac on the right side. On histological analysis, none of the lymph nodes had any location of metastasis, neither micro-metastasis. Myometrial infiltration was 8/19 mm.

**Conclusions:**

Blue dye cervical injection is a “low-cost”, safe, and satisfactory procedure to point out Sentinel Lymph Node of uterus drainage. Other tracers, such as indocyanine green, are widely used in gynecological oncology, but with a higher cost of the product and the needing of a dedicated optical filter to be shown on human view.

**Electronic supplementary material:**

The online version of this article (10.1186/s10397-017-1026-0) contains supplementary material, which is available to authorized users.

## Background

Sentinel lymph node analysis has become a new and innovative method to treat early stages of endometrial cancer [1, 2]. In the aim of reducing complications related to lymphoadenectomy, the use of different tracers to identify first lymph node absorber of the drainage chain is widely propagated in gynecological oncology centers. Indocyanine green cervical injection is considered gold standard for mapping nodes’ drainage, but this technique requires a dedicated optical filter to catch signal. Blue dye is used as a valid alternative (77 Vs 97% bilateral detection rate) in many centers, due to the minor cost of performing. The objective of this video is to prove that methylene blue dye’s cervical injection is a valid and low-cost method to obtain mapping of lymphatic drainage in patient with early endometrial cancer.

## Methods

In a 54-year-old woman, BMI 28, with a recent diagnosis of Endometrial Cancer G2, with no previous imaging suspect of deep myometrial infiltration (> 50%), we performed a radical hysterectomy (class A of Querleu-Morrow’s classification), with bilateral salpingo-oophorectomy. Furthermore, we decided to act cervical injection of blue dye to identify the first lymph node station. We diluted in equal proportions the blue dye and saline and injected 1 cl in depth and 1 cl on the surface of the cervix, at 3 o’clock and 9 o’clock, in order to obtain a precise mapping of the lymphatic course. As in this case, we prefer to practice injection with a spinal anesthesia needle (27G), for the accurate ratio between length and cross section, which makes easier the deep and shallow injection without encountering excessive resistances. Injection of the cervix represents a crucial moment of this method. During this step, indeed, an excess pressure can determine an extravasation of the dye, which would color the entire parametrial space, making it impossible to identify the actual absorber structures. After 20 min, by accessing the retroperitoneum to parametrial level, it was shown with precision the lymphatic course until the first lymph node station from both sides. The lymph nodes had a clear accumulation of tracer, which facilitated the identification even in the absence of appropriate optical filters (Additional file 1).

## Results

One external iliac lymph node and one obturator were resected on the left side and one external iliac on the right side. On histological analysis, none of the lymph nodes had any location of metastasis, neither micro-metastasis. Myometrial infiltration was 8/19 mm. Operation time, including cervical injection, was 47 min, and no abnormal bleedings were reported. Patient was discharged the day after.

## Conclusion

In our experience, blue dye cervical injection is a “low-cost”, safe, and satisfactory procedure to point out sentinel lymph node of uterus drainage. This could help to avoid an overtreatment such as a systematic pelvic lymphadenectomy in patient which have a diagnosis of uterine malignancy. Other tracers, such as indocyanine green, are widely used in gynecological oncology, but with a higher cost of the product and the needing of a dedicated optical filter to be shown on human view. The recent FIRES study demonstrates that indocyanine green cervical injection has a high degree of diagnostic accuracy in patients with endometrial cancer [3]. Is not in the aim of this case report to prove which is the best tracer for endometrial sentinel lymph node, but it is to prove the feasibility of the method with blue dye, a more “comfortable” product.

## Additional file

Additional file 1: Video. (MP4 32,192 kb)
